# Liposome co-encapsulation of anti-cancer agents for pharmacological optimization of nanomedicine-based combination chemotherapy

**DOI:** 10.20517/cdr.2020.87

**Published:** 2021-06-19

**Authors:** Alberto Gabizon, Patricia Ohana, Yasmine Amitay, Jenny Gorin, Dina Tzemach, Lidia Mak, Hilary Shmeeda

**Affiliations:** ^1^Nano-oncology Research Center, Shaare Zedek Medical Center, Jerusalem 9103102, Israel.; ^2^Hebrew University-School of Medicine, Jerusalem 9112102, Israel.; ^3^Lipomedix Pharmaceuticals Ltd., Jerusalem 9139102, Israel.

**Keywords:** Doxorubicin, mitomycin c, prodrug, bisphoshonate, drug delivery, drug carrier, nanoparticle, remote loading

## Abstract

**Aim**: Co-encapsulation of anti-cancer agents in pegylated liposomes may provide an effective tool to maximize efficacy of combined drug therapy by taking advantage of the long circulation time, passive targeting, and reduced toxicity of liposome formulations.

**Methods**: We have developed several liposome formulations of co-encapsulated drugs using various permutations of three active agents: doxorubicin (Dox), mitomycin-C lipidic prodrug (MLP), and alendronate (Ald). Dox and MLP are available in single drug liposomal formulations: pegylated liposomal Dox (PLD, Doxil®), clinically approved, and pegylated liposomal MLP (PL-MLP, Promitil®), in phase 1-2 clinical testing. We have previously shown that co-encapsulation of Dox and Ald in pegylated liposomes (PLAD) results in a formulation with valuable immuno-pharmacologic properties and superior antitumor properties over PLD in immunocompetent animal models. Building on the PLAD and PL-MLP platforms, we developed a new pegylated liposomal formulation of co-entrapped Dox and MLP (PLAD-MLP), with the former localized in the liposome water phase via remote loading with an ammonium alendronate and the latter passively loaded into the liposome lipid bilayer. An alternative formulation of co-entrapped MLP and Dox in which ammonium Ald was replaced with ammonium sulfate (PLD-MLP) was also tested for comparative purposes.

**Results**: PLAD-MLP displays high loading efficiency of Dox and MLP nearing 100%, and a mean vesicle diameter of 110 nm. Cryo-transmission electron microscopy (cryo-TEM) of PLAD-MLP reveals round vesicles with an intra-vesicle Dox-alendronate precipitate. PLAD-MLP was tested in an *in vitro* MLP activation assay with the reducing agent dithiothreitol and found to be significantly less susceptible to thiolytic activation than PL-MLP. Alongside thiolytic activation of MLP, a significant fraction of encapsulated Dox was released from liposomes. PLAD-MLP is stable upon *in vitro* incubation in human plasma with nearly 100% drug retention. In mouse pharmacokinetic studies, PLAD-MLP extended MLP half-life in circulation when compared to that of MLP delivered as PL-MLP. In addition, the MLP levels in tissues were greater than those obtained with PL-MLP, indicating that PLAD-MLP slows down the cleavage of the prodrug MLP to MMC, thus resulting in a more sustained and prolonged exposure. The circulation half-life of Dox in PLAD-MLP was similar to the PLD Dox half-life. The pattern of tissue distribution was similar for the co-encapsulated drugs, although Dox levels were generally higher than those of MLP, as expected from cleavage of MLP to its active metabolite MMC. In mouse tumor models, the therapeutic activity of PLAD-MLP was superior to PL-MLP and PLD with a convenient safety dose window. The Ald-free formulation, PLD-MLP, displayed similar pharmacokinetic properties to PLAD-MLP, but its therapeutic activity was lower.

**Conclusion**: PLAD-MLP is a novel multi-drug liposome formulation with attractive pharmacological properties and powerful antitumor activity and is a promising therapeutic tool for combination cancer chemotherapy.

## Introduction

Formulating nanoparticles containing co-encapsulated drugs is an attractive strategy for co-delivery of drugs with different mechanisms of action and non-overlapping toxicities^[1]^. Conceivably, co-delivery of drugs in the same biodistribution space and with the same time kinetics should optimize *in vivo* synergistic effects of pairs of drugs with a theoretical and/or experimental basis for synergy. Dual or multi-drug liposomes have been initially proposed by the group of Tolcher and Mayer^[2]^ whose approach is based on screening *in vitro* for drug ratios that result in synergistic cytotoxicity. This work has culminated recently in the development of a liposomal formulation of co-entrapped cytarabine and daunorubicin (Vyxeos^TM^) at a specific drug-to-drug ratio which has demonstrated improved survival of patients with acute myeloid leukemia when compared to standard treatment^[3]^ and has been approved by the FDA for clinical use. In another approach to co-encapsulation, we have co-encapsulated an amino-bisphosphonate, alendronate (Ald) with an anthracycline, doxorubicin (Dox), using the former in ammonium salt form to generate a gradient for remote loading of the latter^[4]^. Owing to the immune-modulatory effects of Ald^[5,6]^, this formulation of pegylated liposomal Ald-Dox, abbreviated as PLAD, demonstrates unique properties and superior activity when compared to the standard formulation of pegylated liposomal doxorubicin (PLD) in immunocompetent mouse models.

There is *in vitro* and *in vivo* preclinical evidence of synergy between mitomycin-c (MMC) and Dox^[7-9]^ although the problematic toxicities of these drugs have prevented the use of combination protocols with pharmacologically effective doses. MMC has been shown to suppress P-glycoprotein expression associated with multidrug resistance (MDR1 class), and to sensitize cancer cells to the cytotoxic effects of Dox^[10-12]^. It has also been reported that the number of double-stranded DNA breaks is significantly greater when tumor cells are exposed to a combination of MMC and Dox than to single MMC and may account for the synergistic cytotoxic effect^[13]^. Studies at the University of Toronto have shown that co-loading of MMC and Dox in polymer-lipid hybrid nanoparticles results in enhanced cytotoxic activity and therapeutic efficacy in sensitive tumor cells and MDR tumor cells, bypassing various types of drug efflux pumps^[14-17]^.

The multidrug liposome strategy behind the current study is based on our PLAD technology and on a pegylated liposome formulation of MMC lipidated prodrug (MLP) known as Promitil® and referred here as PL-MLP^[18]^. PL-MLP is highly stable in plasma but readily activated by thiolytic agents in tissues^[19]^. This formulation is currently undergoing clinical testing and has shown, in a recently completed phase 1 dose escalation study, an improved safety profile and higher tolerable dose than the recommended dose of MMC, and a long circulation half-life with minimal or no detectable release of MMC in plasma^[20]^. The aims of the current study were to co-entrap MLP and Dox in a PLAD-based liposome, while retaining the high stability and circulation time characteristics of the PL-MLP and PLD single-drug formulations, and to examine the pharmacologic activity in tumor mouse models of the multidrug liposome versus single drug liposomes. In addition, we also formulated MLP and Dox in a PLD-based liposome using ammonium sulfate instead of ammonium alendronate as Dox loading gradient for comparative purposes.

## Methods

### Liposomes

The multidrug formulation, PLAD-MLP, was prepared, as described by Patil *et al*.^[21]^. We have previously demonstrated that using an ammonium alendronate (250 mmol/L) gradient with pH in the range of 5.5-6.5 results in effective and stable loading of Dox in liposomes^[4]^ similar to that of the classic ammonium sulfate gradient of PLD^[22]^. This gradient is the basis for the previously reported PLAD formulation^[4]^ except that here the histidine buffer was replaced with Hepes buffer. The liposome lipid composition of PLAD-MLP is composed of hydrogenated soybean phosphatidyl-choline (HSPC), methoxy-polyethylene glycol_2000_-distearoyl phosphatidyl-ethanolamine (mPEG_2000_-DSPE), cholesterol and the MLP conjugate at a percent molar ratio of 55:5:30:10, respectively, and is identical to that of PL-MLP^[19]^. We also prepared another multidrug formulation, referred to as PLD-MLP, containing MLP and Dox but no Ald, in which ammonium alendronate was replaced with ammonium sulfate at the same molar concentration. PLAD-MLP and PLD-MLP formulations were adjusted with addition of buffer to a final Dox concentration of 2 mg/mL before use in all experiments. The concentrations of co-encapsulated drugs (MLP, Ald) varied slightly from batch to batch. All lab-prepared liposomes were sterilized by filtration through 0.22 µm-pore cellulose membrane disposable filters.

PL-MLP (Promitil®) was provided by Lipomedix Pharmaceuticals Ltd. (Jerusalem, Israel) and is dispensed in 10 mL-vials for injection containing a sterile suspension of liposomes in phosphate-buffered 5% dextrose at pH 7.0 with a concentration of MLP of 5 mg/mL. For reference, 3.4 mg of PL-MLP contains about 1 mg of MMC-equivalents.

PLD was obtained from commercial sources (Doxil/Caelyx^TM^, Janssen Pharmaceuticals, USA) and is dispensed in 10-mL vials with a concentration of Dox of 2 mg/mL.

PLA-MLP is an intermediate by-product of the PLAD-MLP formulation, obtained by terminating the preparation before the gradient loading of Dox.

The lipid composition of PLAD-MLP (as well as of PLD-MLP) differ from PLAD and PLD due to the presence of MLP at 10% lipid molar ratio at the expense of cholesterol which is reduced to 30%.

Sources of ingredients were Lipoid (Germany) for HSPC and mPEG_2000_-DSPE; Merck (formerly Sigma, St. Louis, MO) for cholesterol; Laurus (Hyderabad, India) for MLP; Teva (Israel) for Ald, Dox, and MMC.

Vesicle size was measured using dynamic light scattering on a Malvern Zetasizer (Malvern, UK). Zeta potential measurements were performed at 25 °C using a Malvern Zetamaster (Malvern, UK). Liposome samples were imaged using (cryo-TEM). Sample preparation and examination by cryo-TEM was carried out at the Hebrew University Center for Nanoscience and Nanotechnology (Jerusalem, Israel), on a FEI Tecnai 12 G2 TEM, operated at 120 kV as previously reported^[23]^. The concentrations of liposomal Dox and MLP were measured by an HPLC method as described in the Supplementary data section.

For a description of methods to determine phospholipid concentration (Bartlett assay), MLP concentration (HPLC assay with UV-detection), plasma stability assays of MLP and Dox, prodrug release assay of MLP, and *in vitro* cytotoxicity assays of MLP and Dox, see Amitay *et al*.^[19]^. For details on the method of Dox measurement, see Shmeeda *et al*.^[4]^. To measure cholesterol concentration, we used an HPLC-based assay with UV detection at 207 nm with minor modifications from a previously described approach^[24]^.

### *In vitro* cell uptake of Dox and MLP

One million tumor cells in 1 mL medium were plated per well in 16-well Petri dishes and incubated with liposomal drugs for 3 h at 37 °C, followed by washing 3 times with phosphate buffered saline. For MLP extraction, adherent cells were digested with isopropyl alcohol overnight at 4 °C, and then MLP concentration was determined by HPLC as previously described^[25]^. For Dox concentration, cells were digested overnight at 4 °C with isopropyl alcohol containing 10% acidified (0.75 N HCl) water, followed by fluorometric analysis of Dox concentration^[21]^. Results of Dox and MLP concentration are in ng/mL and normalized to 1 million cells.

### *In vitro* cytotoxicity studies

The following cell lines were tested: T24 bladder, N87 gastric, IGROV-1 ovarian, HT-29 colon, and human-derived and doxorubicin-resistant human NCI/ADR breast carcinomas. Cytotoxicity studies were performed on cell monolayers in 96-multi well plates by continuous exposure for 72 h to free or liposomal drugs at various concentrations in triplicates. Cell growth rates and IC_50_ values were estimated using a methylene blue colorimetric assay as previously reported^[25]^.

### *In vivo* plasma and tissue bio-analytical assays

Extraction and quantitation of Dox and MLP from plasma and tissues were done following previously published methods^[19,26]^. Because of the rapid covalent binding of MMC with DNA and other biomolecules and/or tissue metabolism, we were not able to quantitate MMC in cells or tissues. In tissue distribution studies, the tissue sampling times were 24 and/or 48 h after injection when the liposome blood levels have gone significantly down and a large fraction of the injected dose has already been distributed to tissues. In animals injected with free Dox, sampling was 2 h after injection since the free drug distributes very quickly and reaches maximal concentration in tissues shortly after injection.

### Animals and treatments

Female BALB/c and Sabra, 8-11 weeks old, were obtained from Harlan Biotech (Jerusalem, Israel). Experiments in mice were performed either at the Shaare Zedek Medical Center Animal Lab or at the Animal Facility of the Hebrew University-Hadassah Medical School. All animal experiments were approved by the Animal Ethics Committee of the Hebrew University - Hadassah Medical School. In *in vivo* studies, the following BALB/c mouse tumor models were tested: 4T1, C26, and M109 sensitive and resistant tumors. For the 4T1 model, a triple receptor-negative, breast cancer mouse (BALB/c) model was used. One-hundred thousand tumor cells were inoculated into the mouse hind footpad, and when tumors were detectable (~2 mm size), treatment was initiated by the intravenous route. Three intravenous treatments were administered at weekly intervals, and mice were followed individually for tumor growth by measuring the thickness of the injected footpad with calipers. In the C26 model, 1 million tumor cells per mouse were inoculated intraperitoneally, followed 5 days later by intravenous weekly treatments with free or liposomal drugs. Weight curves and survival were recorded. In the M109 model, 1 million tumor cells were inoculated into the mouse hind footpad, followed 5-7 days later by intravenous weekly treatments with free or liposomal drugs. For treatment with multidrug liposomes, the dose injected was based on the Dox concentration. When combining 2 single drug liposomes, each was given at doses equivalent to those administered in the co-encapsulated formulation. For single drug liposome treatment, the doses injected were the pharmacologically optimal doses as determined previously in our laboratory. Mice were followed individually for tumor growth by measuring the thickness of the injected footpad with calipers. Mice with tumors greater than 5 mm were culled. Experiment follow-up lasted between 1 and 3 months.

### Statistical analysis

The significance of *in vitro* and *in vivo* results was analyzed by the Student’s *t*-test and the log-rank test using Prism, version 8.0 (Graphpad software, San Diego, CA, USA). The *P*-values are two-sided and considered non-significant when greater than 0.05.

## Results

### Formulations

The liposomes tested in this study included two control single drug formulations, our experimental multi-drug formulation (PLAD-MLP), and three more dual drug formulations for comparison purposes.

**Figure 1:**
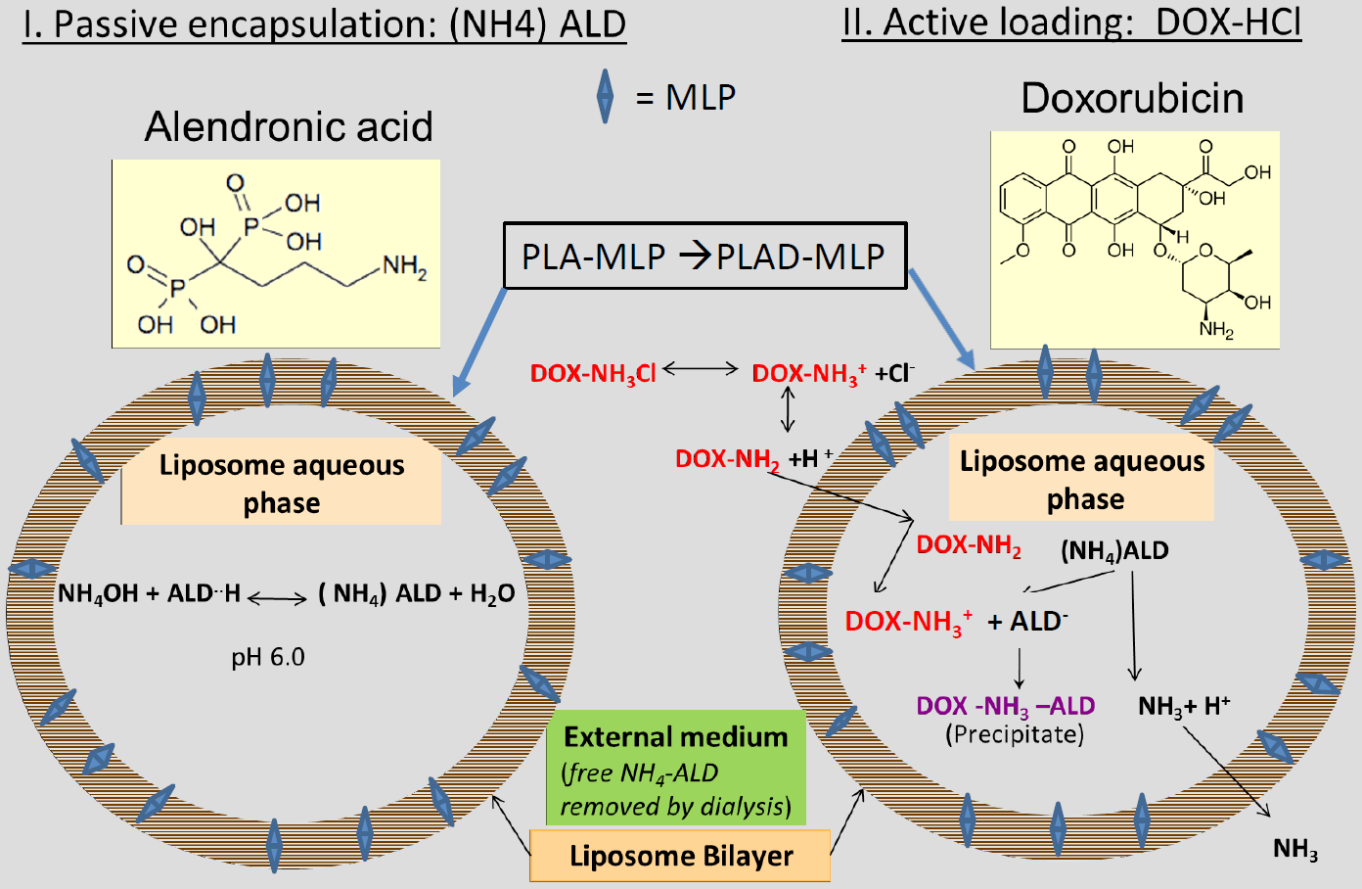
Schematic diagram of PLAD-MLP, a liposome formulation with co-encapsulated Ald, Dox and MLP (prodrug of MMC). A liposome formulation with MLP in the lipid bilayer and ammonium (NH_4_)Ald in the water phase is remote loaded with Dox, resulting in a multidrug liposome co-encapsulating two potent cytotoxic agents, and an immunomodulatory aminobisphosphonate (Ald). (NH_4_)Ald can be replaced with ammonium sulfate without affecting the remote loading of Dox

- PLD, an approved product in extensive clinical use containing as active pharmaceutical ingredient a salt of Dox sulfate in the aqueous phase mostly present in rod-like precipitates^[27,28]^.
- PLAD or pegylated liposomal alendronate salt of doxorubicin, a dual drug formulation with chemotherapeutic and immunotherapeutic activities previously characterized and reported^[4]^.
- PLAD-MLP, a multi-drug liposome with co-encapsulated MLP in the lipid bilayer and Dox remote-loaded in the water phase using ammonium alendronate gradient. A diagram depicting the 2-step loading process using the ammonium alendronate gradient is shown in Figure 1.
- PL-MLP, an experimental formulation in clinical trials, with a prodrug, MLP, inserted in the lipid bilayer and activated to MMC by thiolytic cleavage^[19]^.
- PLD-MLP, an Ald-free formulation similar to PLAD-MLP, with co-encapsulated MLP and Dox remote loaded with an ammonium sulfate gradient.
- PLA-MLP, a formulation similar to PL-MLP but containing an ammonium Ald salt at 250 mmol/L and pH ~6.0 in the water phase.

The physico-chemical characteristics of a representative batch of PLAD-MLP and other formulations are presented in Table 1 for comparison.

**Table 1:** Characteristics of the PLAD-MLP multidrug liposome and related liposomes^1^

Formulation	Phospholipid (µmol/mL)	Cholesterol (mg/mL)	MLP (mg/mL)	Dox^2^ (mg/mL)	Ald (µmol/mL)	Mean Diameter (nm)	PDI	Zeta potential (mV)
PLAD-MLP	19.7	2.3	2.4	2.0	2.9	119	0.057	-15.0
PL-MLP	26.7	5.4	5.0	n/a	n/a	102	0.085	-13.1
PLD	12.4	3.4	n/a	2.0	n/a	83	0.049	-14.8
PLAD	16 .3	4.5	n/a	2.0	3.7	83	0.069	-14.9
PLD-MLP	20.6	3.1	2.2	2.0	n/a	129	0.049	Not done

^1^Three or more batches of each formulation were prepared for this study; ^2^Dox concentration adjusted to 2 mg/mL ± 10% in final product. MLP: Mitomycin-C lipidic prodrug; PLAD: Dox and Ald in pegylated liposomes; PLD: pegylated liposomal doxorubicin

PLAD-MLP had a final Dox concentration adjusted to 2 mg/mL, and a final MLP concentration ranging between 2.5-3.0 mg/mL, resulting in an MLP/Dox molar ratio of ~0.7. The loading Ald/Dox molar ratio was 1 and the final Ald/Dox achieved was between 1 and 1.5. The nanoparticle size of PLAD-MLP was between 110-130 nm, slightly larger than that of PL-MLP. Polydispersity index was narrow (≤ 0.15). The percent entrapment efficiency was determined by calculating the MLP/cholesterol ratio at input and at completion of formulation, and the Dox/phospholipid ratio prior to Dox loading and after removal of free (unencapsulated) Dox. The entrapment efficiency was nearly 100% for MLP, and 80%-90% for Dox. There was a loss of ~20% of lipids during the formulation process in the small-sized batches (< 50 mL) prepared in the lab, mainly in the steps of extrusion, and dialysis/diafiltration, as determined by phospholipid analysis. The ammonium sulfate and ammonium alendronate gradients were equally effective in terms of the final loading of Dox and other liposome properties [Table 1].

Column chromatography of the final PLAD-MLP formulation showed essentially all drug in the void volume together with liposomes and no free Dox, MLP, or MMC in late fractions (data not shown).

**Figure 2:**
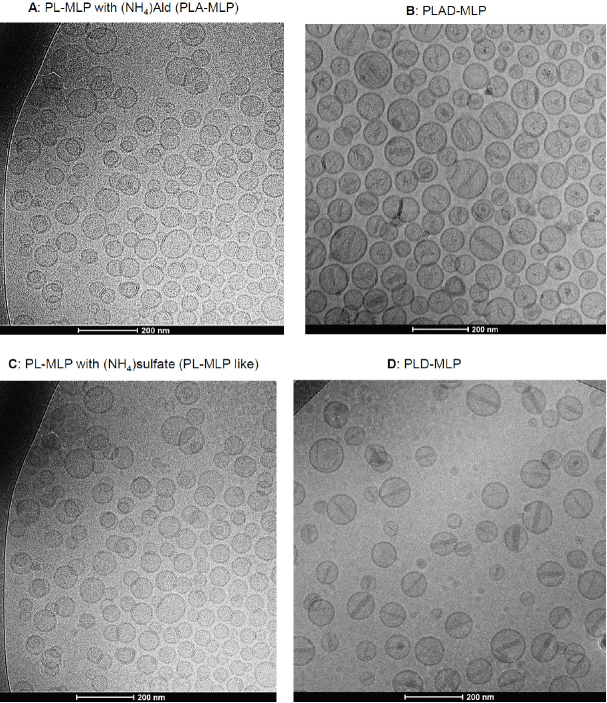
Cryo-transmission electron microscopy images of PLAD-MLP liposomes and related formulations. A, B: PL-MLP liposomes with ammonium Ald in the water phase before (A: PLA-MLP) and after (B: PLAD-MLP) Dox loading. Note the disorganized precipitates of Ald-Dox with few cases of rod formation; C, D: PL-MLP liposomes with ammonium sulfate in the water phase before (C: PL-MLP like) and after (D: PLD-MLP) Dox loading. Note the well-formed rods across the entire liposome

PLAD-MLP was examined by cryo-TEM and compared to other formulations [Figure 2A-D]. A dominant population of spheroidal vesicles which are mostly unilamellar liposomes is present. Unlike PL-MLP, no disks were observed in PLAD-MLP suggesting that the Ald salt in the inner water phase helped stabilize vesicular formation. Precipitated crystals of doxorubicin salt in the interior water phase of the vesicles are apparent. The precipitate formations were often disorganized and variable in shape and appearance, and sometimes organized as the rod-like formations previously reported for PLD^[29]^ [Figure 2A-D]. As in PLAD liposomes, the intra-vesicle rods of PLAD-MLP were apparently shorter than those of PLD since they did not reach the inner side of the bilayer. In contrast to PLAD-MLP, the rods of PLD-MLP were longer, reaching the bilayer with frequent oval elongation of the liposome, as known for PLD. This indicates that the Ald-Dox precipitates and rods have different characteristics from those described for the sulfate-Dox crystalline precipitates in the PLD clinical formulation^[30]^. Some of the PLAD-MLP and PLD-MLP liposomes show electron-dense bands in the bilayer which most likely represent MLP molecules undergoing stacking into distinct membrane domains as reported previously for PL-MLP^[23]^.

### Prodrug cleavage and activation assay

**Figure 3:**
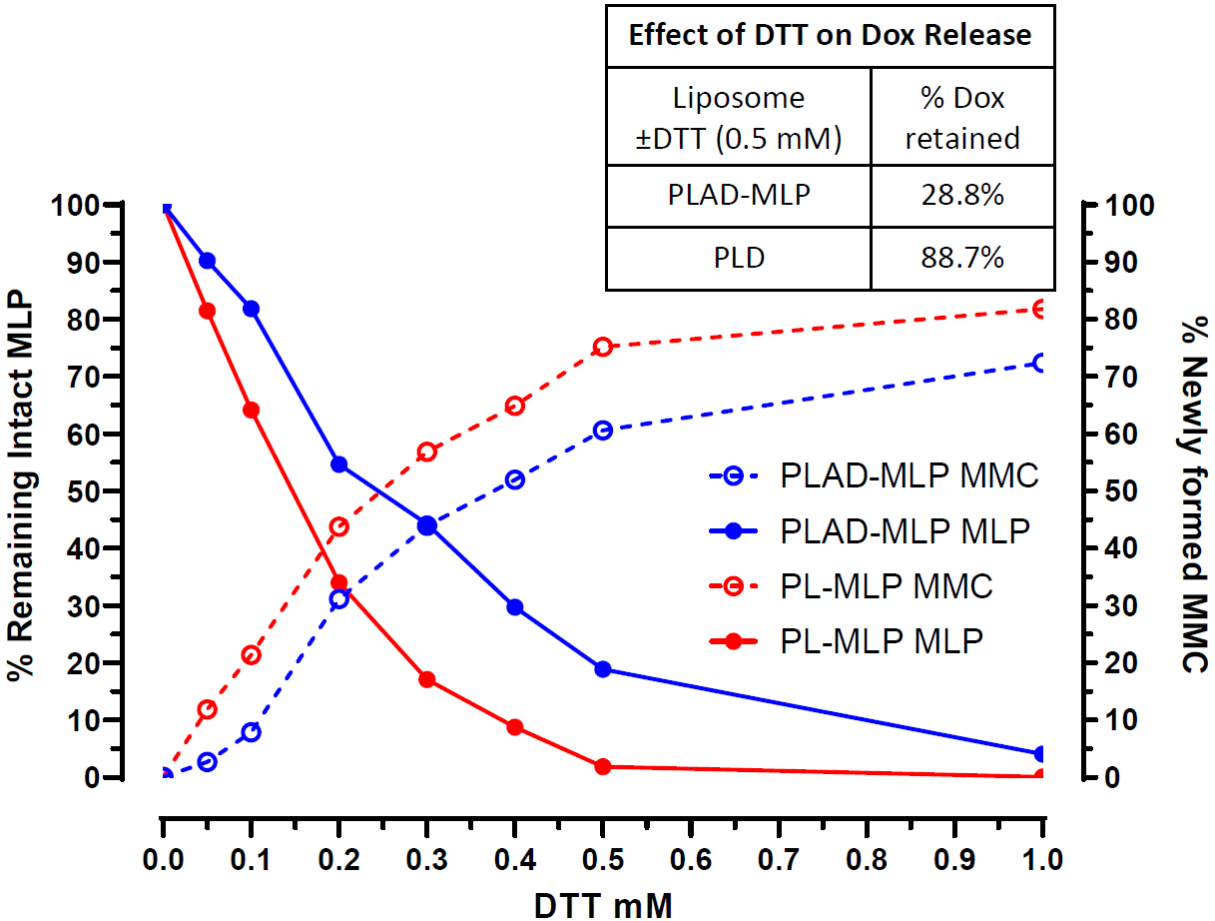
Kinetics of MLP cleavage and MMC release by DTT. PL-MLP and PLAD-MLP (0.2 mg/mL) were incubated with DTT at 37 °C for 1 h. Samples were then processed and run on HPLC. Note the slower cleavage of MLP and lower rise of MMC for PLAD-MLP, particularly when at DTT concentrations under 0.5 mmol/L. The inset table shows a strong effect of DTT on release of Dox from PLAD-MLP, while, in the case of PLD, DTT-induced Dox release is minimal, suggesting that MLP cleavage increases the liposome permeability and causes Dox leakage

In this assay, dithiothreitol (DTT), a potent reducing agent, cleaves PL-MLP activating the prodrug and releasing free MMC. A DTT:MLP 1:1 molar ratio is required for 90-100% cleavage of MLP within an incubation time of 30-60 min^[19]^. The MLP drug release assay for PL-MLP and PLAD-MLP [Figure 3] shows the DTT concentration dependence of MLP activation at 37 ºC (*i.e.*, cleavage and release of free MMC). In the case of PLAD-MLP, the data show that, when incubated for 1 h at 1:1 molar ratio, DTT was not able to completely cleave MLP and release MMC, as predicted by a simple stoichiometric reaction, suggesting that the liposomes with co-encapsulated MLP and Dox are more resistant to thiolytic cleavage than PL-MLP. Therefore, we conclude that there is no risk of premature activation of the prodrug in the PLAD-MLP formulation.

The thiolytic activation of MLP causes transient liposome instability^[19]^ which results in collapse of the gradient and release of a significant fraction of encapsulated Dox from the liposomes [Figure 3 and inset Table]. This may facilitate simultaneous release of both active drugs in the tumor site.

### Stability in human plasma of liposome co-encapsulated drugs

**Figure 4:**
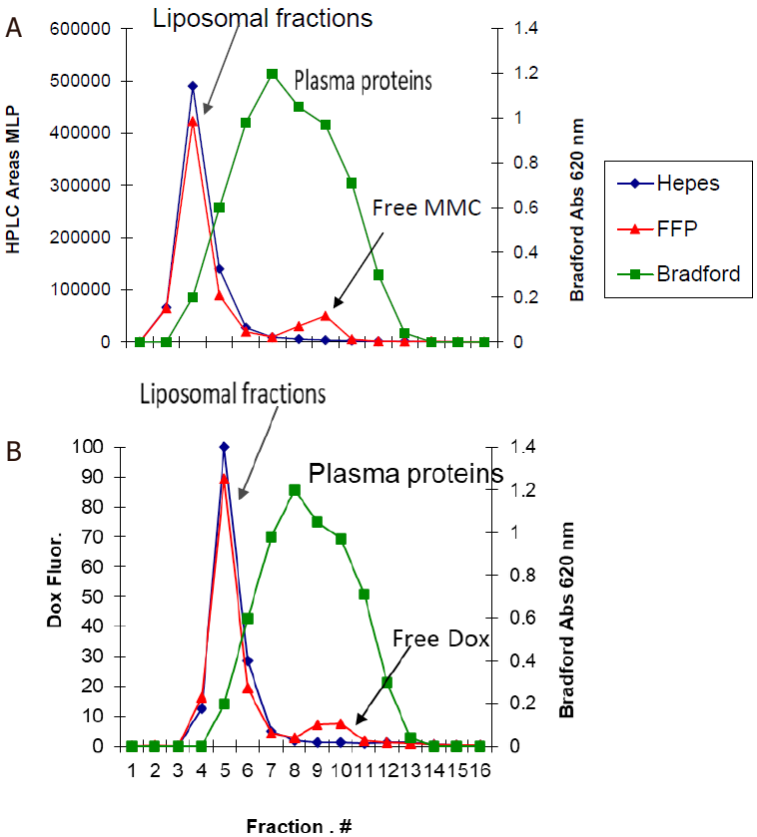
Plasma stability of PLAD-MLP. Gel chromatography elution profile of PLAD-MLP incubated at 37 °C for 2 h in 80% plasma. Liposomes elute in fractions 4-7; plasma proteins (green) elute mostly in fractions 8-11; free drugs elute in fractions 12-14. A: HPLC determination of MLP (red for plasma, dark blue for buffer) indicates that it remains nearly 100% intact and liposome associated during exposure to 80% human plasma for 2 h at 37 °C; B: fluorometric determination of Dox (red for plasma, dark blue for buffer) indicates that Dox remains nearly 100% liposome associated during exposure to 80% human plasma for 2 h at 37 °C. FFP: fresh frozen human plasma

The plasma stability of PLAD-MLP liposomes was assessed as described in Methods. Figure 4A shows the release of MLP in fractions of eluent collected from the column after incubation of the liposomes in human plasma and in buffer. Figure 4B is a similar graph showing release of doxorubicin in the eluent fractions after incubation of the liposomes in human plasma and in buffer. Nearly all the MLP and Dox remain liposome-associated and elute together with liposomes in fractions 4-6, while plasma proteins elute mostly in fractions 7-11. There was a very small fraction of MLP and Dox partitioning into plasma proteins and there was no detectable drug release in buffer. This indicates that drug leakage, exchange with proteins, and/or cleavage to free mitomycin C in plasma are minimal and probably insignificant.

### *In vitro* tumor cell uptake of liposome co-encapsulated drugs

The liposomal formulations were incubated for 3 h with the selected cell line at a doxorubicin concentration of 6.5 µmoles/L (3.7 µg/mL) and final MLP concentration of 5 µmoles/L (5.7 µg/mL).

**Figure 5:**
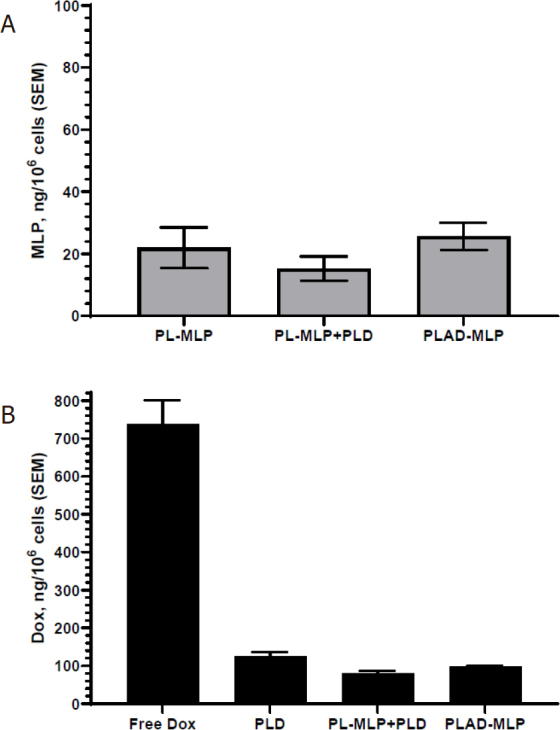
*In vitro* liposomal drug uptake by T24 cells. Cells were incubated for 3 h in the presence of 10 µmol/L Dox and/or 7 µmol/L MLP in various liposomal formulations. A: uptake of MLP; B: uptake of Dox, including free Dox as control. Note that low and comparable levels of the liposomal drugs drug are taken up by tumor cells

There is no significant drug leakage or prodrug cleavage in culture medium during this time interval (data not shown), so that the uptake depends on liposome endocytosis which is limited with pegylated liposomes. Results for the T-24 bladder cancer cells are shown in Figure 5. Exposure to PLAD-MLP resulted in low cell uptake for both doxorubicin and MLP, with comparable values to PLD and PL-MLP. The drug uptake was slightly lower when cells were exposed to a combination of PLD and PL-MLP [Figure 5], probably owing to the larger (2x) number of liposomes per cell competing for uptake. When Dox and MLP cell levels are compared, the Dox levels are much greater indicating that a large fraction of MLP is cleaved after cell uptake and generates active MMC.

### *In vitro* cytotoxicity of liposome co-encapsulated drugs

**Figure 6:**
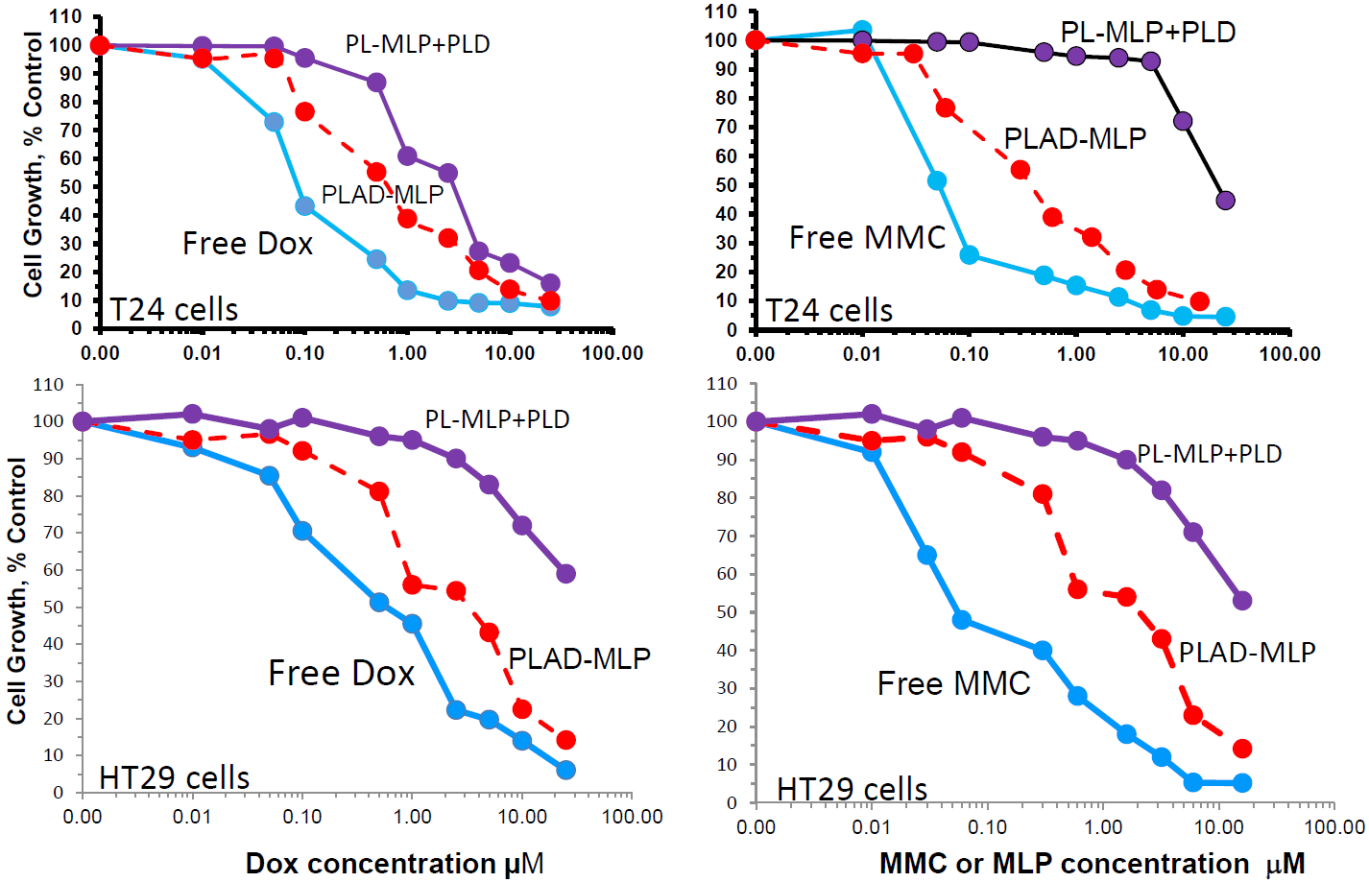
*In vitro* cytotoxicity of PLAD-MLP and comparators. Tumor cells were seeded in 96-multiwell plates. After overnight incubation, the liposomal drugs were added in triplicates for each concentration and cells were incubated further for 72 h. Cell growth was evaluated colorimetrically with methylene blue staining and growth rates were calculated as previously described^[25]^. Upper and lower panels show results for Dox and MLP/MMC cytotoxicity in T24 human bladder tumor cells and HT29 human colon tumor cells, respectively. Note the greater cytotoxicity of the multidrug formulation, PLAD-MLP (red lines), over the combined addition of PL-MLP and PLD at equal drug concentrations. PLAD-MLP values were significantly different from other liposome formulations (paired *t* test, *P* < 0.05)

The cytotoxicity of the PLAD-MLP multidrug liposomal formulation in comparison to free drugs and related liposomal formulations was examined in various human tumor cell lines [Figure 6]. Previous studies have shown that the high stability of pegylated formulations and minimal liposome uptake by most types of tumor cells in *in vitro* tissue culture results in substantially reduced cytotoxicity when compared to free drugs^[31]^. Since the tumor cell uptake of PLAD-MLP is as low as that of PLD and PL-MLP, as shown by the drug levels presented in Figure 5, and liposomal Ald (*i.e.*, PLA) has negligible cytotoxicity *in vitro* on tumor cell lines^[6]^, we would expect the cytotoxic effect of PLAD-MLP to be similar to that of the combination of PLD and PL-MLP. However, surprisingly, the cytotoxic effect of the multi-drug formulation was consistently greater in the various tumor cell lines tested (T24, N87, and HT29). The IC_50_ values of the multidrug formulation are several-fold lower than for the combination of PLD and PL-MLP, implying a synergistic effect when the kinetics of drug release are controlled by a single carrier. While we took care to titrate the concentrations of PLD and PL-MLP to the contents of Dox and MLP in the multidrug formulation, the cell uptake and breakdown of two liposomes may be less effective or take longer than that of a single liposome delivering the same drug payload, thereby accounting for the higher cytotoxicity of PLAD-MLP. PLAD-MLP showed greater cytotoxicity than PLD and PL-MLP alone or combined. In some cell lines, PLAD-MLP approximated the cytotoxic activity of free Dox and free MMC, despite the low cell uptake of these pegylated liposomal drugs *in vitro* [Figure 5]. Two representative examples of the cytotoxicity experiments are presented in Figure 6, showing superior cytotoxicity of PLAD-MLP over PL-MLP and PLD on T24 cells [Figure 6A and B] and over a combination of PLD and PL-MLP on HT29 cells [Figure 6C and D]. Similar results were obtained with other cell lines [Supplementary Table 1].

We also tested two more formulations in the cytotoxicity assay: PLA-MLP (PL-MLP loaded with ammonium Ald in the water phase without Dox) and PLD-MLP (PL-MLP loaded with Dox sulfate without Ald). PLA-MLP cytotoxicity was low and similar to that of PL-MLP. PLD-MLP cytotoxicity was higher and comparable or slightly lower than that of PLAD-MLP [Supplementary Figure 1].

**Figure 7:**
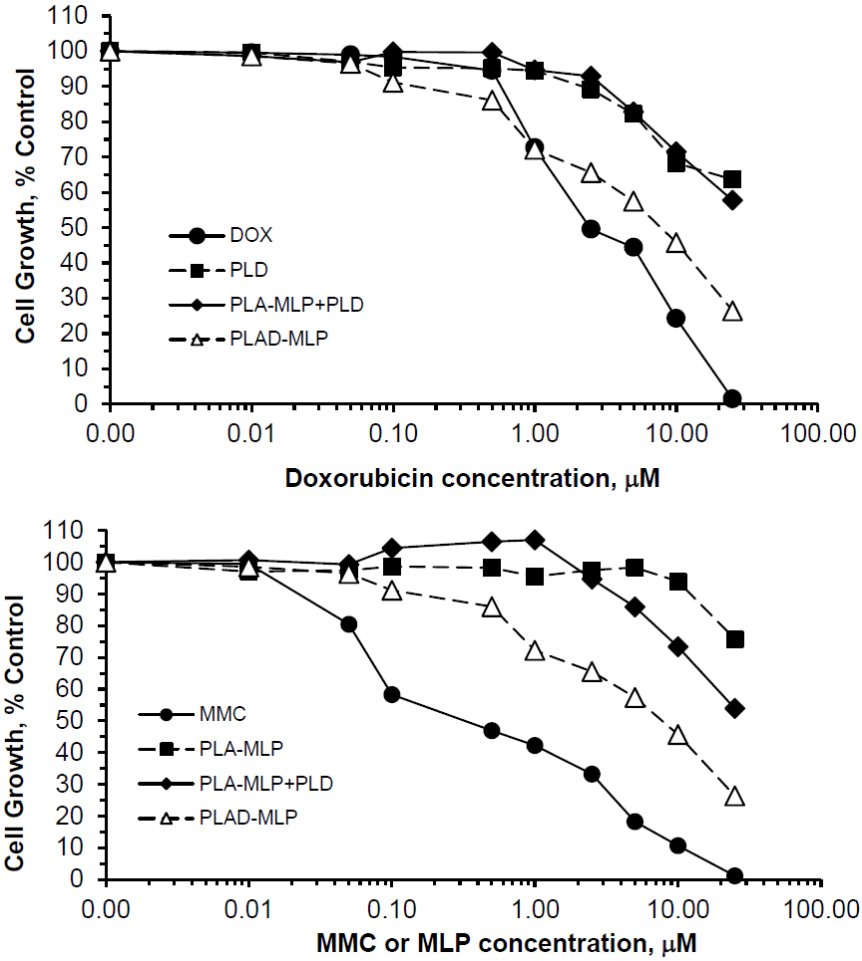
*In vitro* cytotoxicity of PLAD-MLP and comparators on the multidrug-resistant, NCI/ADR tumor cell line. Identical experiment as described in Figure 6, except for the use of the NCI/ADR human cell line. In this experiment, we used PL-MLP loaded with Ald (=PLA-MLP) as control to rule out any contribution of Ald to the enhanced cytotoxic effect of PLAD-MLP. Note the greater cytotoxicity of MMC as compared to Dox, as expected from the MDR-1, P-glycoprotein mediated mechanism of resistance which spares MMC. Note, as well, the greater cytotoxicity of PLAD-MLP over a combination of PLA-MLP and PLD. PLAD-MLP values were significantly different from other liposome formulations (paired *t* test, *P* < 0.05)

Significantly enhanced cytotoxicity was demonstrated with PLAD-MLP on doxorubicin resistant (NCI/ADR) cells, when compared to cells incubated with PLD, PLA-MLP, and a combination of the latter two [Figure 7]. These *in vitro* results suggest synergistic cytotoxicity of MLP and doxorubicin when co-encapsulated in the same liposome vehicle and highlight the ability of this multidrug formulation to overcome Dox resistance.

### Pharmacokinetics and biodistribution of liposome co-encapsulated drugs

**Figure 8:**
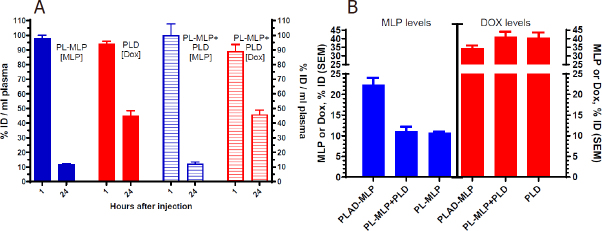
Plasma levels of liposomal drugs at 1 and 24 h after intravenous administration. A: drug plasma levels after single administration or co-administration of PL-MLP and PLD in BALB/c. No change in drug levels at 24 h, indicating no pharmacokinetic interference between PL-MLP and PLD under the experimental conditions; B: comparison of drug plasma levels 24 h after injection of PLAD-MLP, PL-MLP, PLD, or PL-MLP+PLD. There is a major (> 2-fold) increase of MLP levels when drugs are co-encapsulated in PLAD-MLP, but no change of Dox levels, as compared to single liposome (PL-MLP) or combined liposomes (PL-MLP+PLD) co-administration

We first tested the blood clearance of separately and co-administered PL-MLP and PLD and found that the 24-48 h plasma levels were comparable and clearly not affected by co-administration of two formulations [Figure 8A], suggesting that there was no significant pharmacokinetic interference at the dose tested between the single drug liposomes. As expected from previous studies^[19]^, PL-MLP was cleared faster than PLD. Cleavage of MLP from circulating liposomes by the reticulo-endothelial system and endothelial cells through membrane-associated protein disulfide isomerases has been invoked as a possible mechanism^[19]^.

We then tested the pharmacokinetic profile of the multidrug liposome, PLAD-MLP. We found that the MLP/Dox ratio of PLAD-MLP drops by approximately 30% after 24 h indicating that MLP is cleared somewhat faster than Dox. However, as seen in Figure 8B, the amount of MLP in plasma 24 h after administration of PLAD-MLP is about 2-fold higher than after administration of PL-MLP with or without co-injection of PLD. In contrast, Dox levels were similar for PLAD-MLP and PLD. Thus, co-encapsulation reduces the rate of clearance of MLP prolonging its half-life, while leaving the clearance of Dox unchanged. This unexpected observation indicates that the presence of the Dox-Ald salt stabilizes MLP in the bilayer or reduces liposome interaction with endothelial surfaces to prevent early cleavage of the prodrug from circulating liposomes.

**Figure 9:**
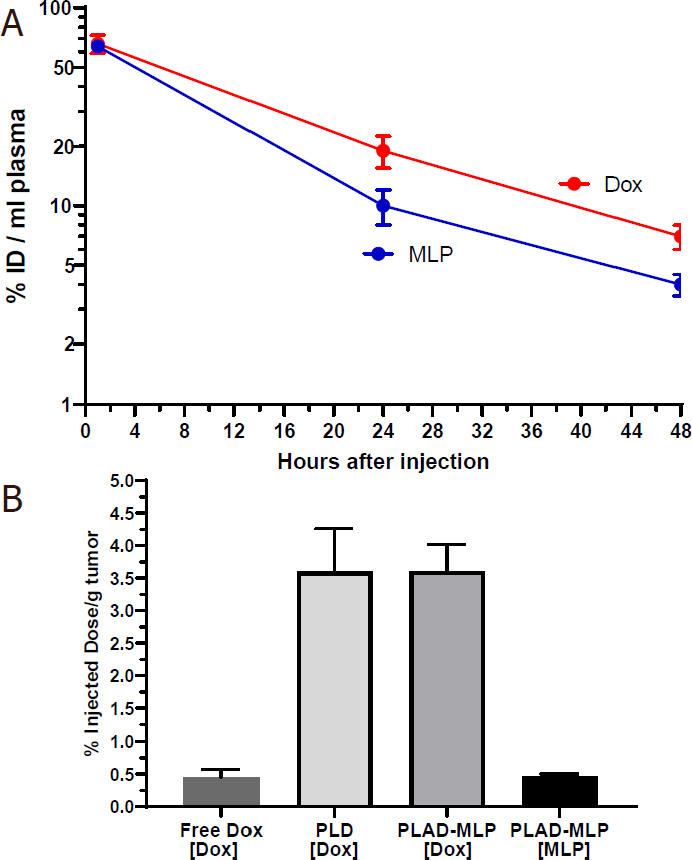
A: plasma clearance of PLAD-MLP in M109-tumor bearing BALB/c mice; B: tumor levels of PLAD-MLP and PLD in M109 tumor-bearing mice (48 h and 2 h post-injection for liposomal drugs and free drug respectively)

**Figure 10:**
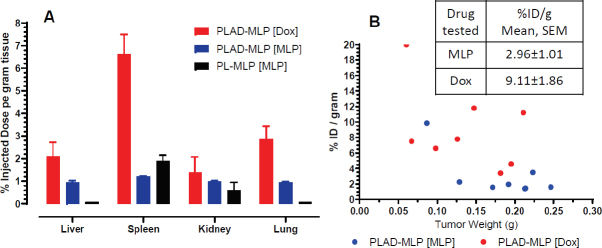
Biodistribution of PLAD-MLP in mice. Drug levels measured 24 h after injection to M109 tumor-bearing BALB/c mice. A: normal tissue levels after injection of PLAD-MLP or PL-MLP; B: tumor individual measurements after injection of PLAD-MLP.

We then tested plasma clearance and tissue distribution of PLAD-MLP in BALB/c mice bearing subcutaneous implants of the M109 tumor [Figures 9 and 10]. We confirmed that MLP was cleared faster than Dox [Figure 9A]: at 24 h after injection, the MLP/Dox molar ratio went down by 33% (*i.e.*, from 0.645 before injection to 0.43 at 24 h). The gap in plasma levels did not widen any further between 24 h and 48 h after injection. At 48 h, the tumor levels of Dox in mice injected with PLAD-MLP were comparable to those observed after injection of PLD and at least 10-fold greater than those obtained after injection of free Dox [Figure 9B]. Tumor Dox levels were far higher (~7-fold) than MLP levels. Since Dox plasma levels at 48 h were only 2-fold greater than MLP levels, this suggests that MLP has been partly cleaved and/or cleared from the tumor tissue.

In a separate experiment, we tested PLAD-MLP tissue distribution in comparison to PL-MLP in liver, spleen, kidney, lungs, and heart, as well as in tumor tissue of tumor-bearing mice [Figure 10]. Comparison of MLP and Dox tissue levels after injection of PLAD-MLP showed relatively greater Dox values [Figure 10A], as expected, given the rapid tissue cleavage of MLP particularly in liver and lungs, as previously reported^[19]^. Interestingly, in PLAD-MLP injected mice, low levels of MLP were often detectable after 24 h in liver and lung whereas in PL-MLP injected mice, we were never able to detect MLP in these tissues [Figure 10A], suggesting that the *in vivo* cleavage of MLP is slower when the prodrug is delivered in the multi-drug formulation, a finding consistent with the results of the *in vitro* prodrug cleavage assay mentioned above. In the tumor tissue, Dox levels were significantly greater than MLP when expressed as percent injected dose per gram (%ID/g) [Figure 10B], as observed in normal tissues, indicating that MLP becomes bioavailable in tumor tissue. The levels of MLP were still far greater in tumor than what we have found for PL-MLP^[19]^, probably owing to the longer circulation time and thereby greater enhanced permeability and retention effect of PLAD-MLP. There was a trend to higher liposomal drug uptake per gram tumor in smaller tumors [Figure 10B] in agreement with general observations for liposomes in animals and humans^[32]^.

### Therapeutic efficacy

Initially, we tested for *in vivo* toxicity in BALB/c tumor-free mice with 3 intravenous injections of PLAD-MLP and PLD-MLP on days 1, 8, and 22, and followed the animals for 60 days [Supplementary Figure 2]. The dose per injection was 10 mg/kg Dox which is the maximal weekly safe dose of PLD. This resulted in a concomitant dose of 13-16 mg/kg MLP which is 40%-50% of the maximal weekly safe dose of PL-MLP, and 5 mg/kg Ald. All 4 mice in each group survived. One mouse in the PLAD-MLP group lost 15% weight after the second injection but recovered promptly [Supplementary Figure 2]. An attempt to raise the dose of Dox to 15 mg/kg with a concomitant proportional raise of the dose of co-encapsulated drugs resulted in death within 2 weeks. Altogether, the mouse toxicity studies indicate that, when Dox is co-encapsulated in PLAD-MLP, the maximal subtoxic combination dose with MLP is 8-10 mg/kg Dox and 10-15 mg/kg MLP. When Dox is encapsulated as single agent (PLD) or together with alendronate (PLAD), the optimal subtoxic dose of Dox is 8-10 mg/kg, nearly equal to that of PLAD-MLP.

*In vivo* therapeutic studies were conducted in tumor-bearing mice to compare the efficacy of liposome co-encapsulated drugs with that of free drugs, liposomal single drugs, and their combination. Since the effects of liposome-encapsulated Ald, one of the components of PLAD-MLP, are mediated by the immune system^[6]^, we chose to focus here on syngeneic tumor models in immunocompetent mice for efficacy studies.

**Figure 11:**
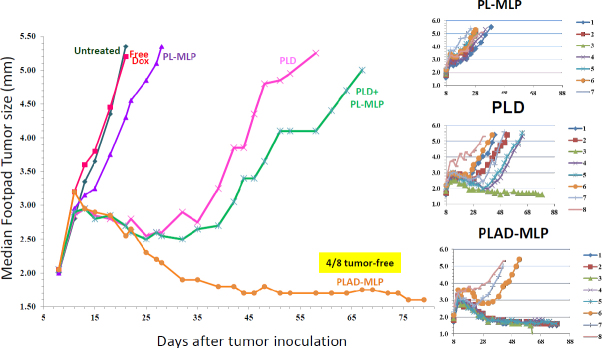
Superior therapeutic activity of PLAD-MLP in the 4T1 tumor model (triple negative mouse breast carcinoma). BALB/c female mice were inoculated with 10^5^ tumor cells subcutaneously in the right rear footpad. Treatment given intravenously on days 8 and 15. When Dox and MLP are co-encapsulated, the doses are shown as Dox/MLP respectively. *N* mice/group=5-8. Dose (1st → 2nd): PLD, 10 mg/kg → 8 mg/kg; PL-MLP, 35 mg/kg → 30 mg/kg; PLD+PL-MLP, 10 mg/kg + 16.8 mg/kg → 8 mg/kg + 13.5 mg/kg; PLAD-MLP, 10/16.8 mg/kg → 8/13.5 mg/kg. The median tumor size values for the PLAD-MLP were significantly different when compared to other groups (Wilcoxon matched-pairs signed rank test, *P* < 0.05)

In the 4T1 tumor model, the following drug formulations were tested: free Dox, PL-MLP, PLD, PLAD-MLP, and a combo of PL-MLP and PLD. Consistent with our prior experience in various tumor models with free drugs, the activity of free Dox in the 4T1 tumor model is minimal with no detectable tumor growth delay and no tumor regression. When the tumor size growth curves are compared, based on median values per group, there was a clear superiority of the multi-drug PLAD-MLP formulation over all other groups in terms of tumor growth delay and apparent cure rate (50%) [Figure 11]. This is despite the fact that single agent PL-MLP did not have any significant antitumor activity. There was no significant toxicity detected in any of the groups in this study as indicated by the body weight curves and lack of toxic deaths (data not shown). When PLAD-MLP was compared to PLAD in the 4T1 tumor model, both treatments were equally effective and significantly superior to PLD [Supplementary Figure 3]. Collectively, the data indicate that, in the 4T1 tumor model, encapsulated Ald plays an important role in the therapeutic outcome.

**Figure 12:**
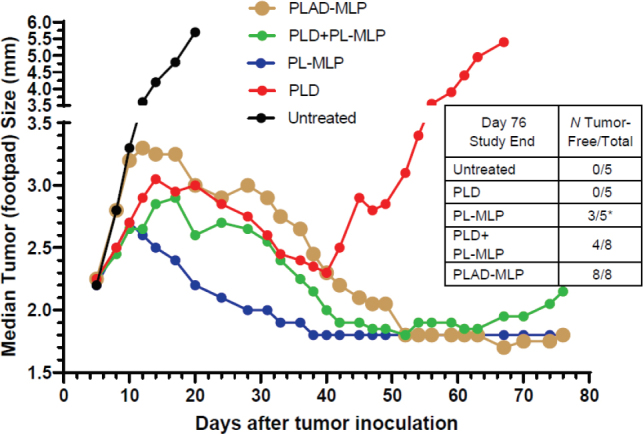
Superior therapeutic activity of PLAD-MLP in the M109 tumor model (mouse lung carcinoma). BALB/c female mice were inoculated with 10^6^ tumor cells subcutaneously in the right rear footpad. Treatment given intravenously on days 5 and 12. When Dox and MLP are co-encapsulated, the doses are shown as Dox/MLP respectively. *N* mice/group = 5-8. Dose (1st → 2nd): PLD, 10 mg/kg → 8 mg/kg; PL-MLP, 35 mg/kg → 30 mg/kg; PLD+PL-MLP, 10 mg/kg + 13 mg/kg → 8 mg/kg + 10 mg/kg; PLAD-MLP, 10/13 mg/kg → 8/10 mg/kg. Statistical analysis of last 10 time points (Wilcoxon matched-pairs signed rank test) of PLAD-MLP vs the following groups: PLD+PL-MLP, *P* = 0.004; PLD, *P* = 0.002; PL-MLP, *P* non-significant

In the M109 tumor model, PLAD-MLP was superior to all other treatments when considering the median tumor size curve, the number of animals (100%) with cures or complete tumor regression, and the absence of toxicity [Figure 12 and inset Table]. PL-MLP at a high dose of 35 mg/kg was also very effective and did achieve complete tumor regression in all mice but at the cost of severe lethal toxicity. We did not test free drugs in this model since we have previously shown that free Dox and free MMC are therapeutically inferior to the liposome formulations^[33,34]^.

**Figure 13:**
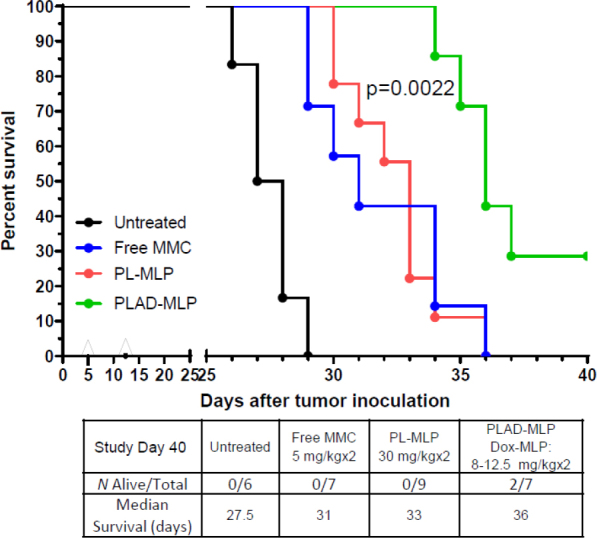
Superior therapeutic activity of PLAD-MLP in the C26 tumor model (mouse colon carcinoma). BALB/c female mice were inoculated with 10^6^ tumor cells intraperitoneally. Treatment given intravenously on days 5 and 12. *N* mice/group = 6-9. For dose, see inset Table. Statistical analysis (log rank test) of PLAD-MLP *vs*. the following groups: Untreated, *P* = 0.0002; MMC, *P* = 0.0061; PL-MLP, *P* = 0.0022

In the mouse C26 carcinoma tumor model of lung metastases, tumor cells were inoculated intravenously, and the drug formulations tested were free MMC, PL-MLP, PLA-MLP, and PLAD-MLP. Results are shown in Figure 13 as a survival curve and analyzed by the log rank test. Median survivals of the different groups are presented in Figure 13 inset Table. There were no apparent toxic effects due to treatment with any of the liposome formulations, with the exception of one mouse in the PLAD-MLP group that was sacrificed on day 18. Mice treated with PLAD-MLP had the longest median survival at 36 days. The co-encapsulation of MLP and Ald in PLA-MLP did not improve efficacy over PL-MLP indicating that the full multidrug cocktail is required for optimal efficacy. Statistical analysis confirmed that the therapeutic effect of the co-encapsulated formulation was significantly superior to other treatment groups based on the survival time (see legend of Figure 13).

**Figure 14:**
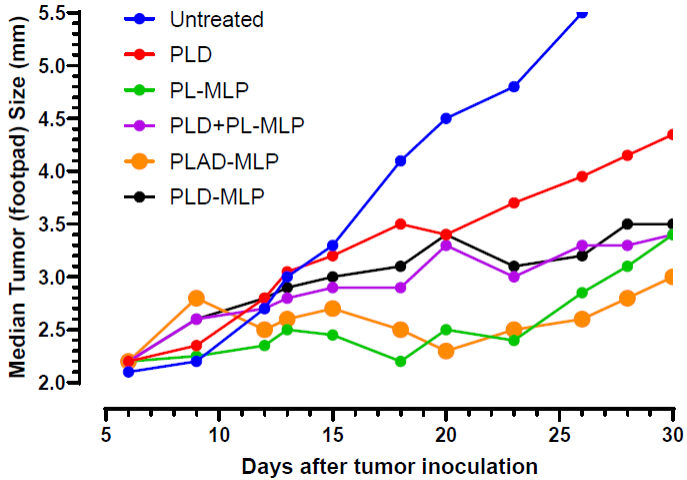
Superior therapeutic activity of PLAD-MLP in the M109R tumor model (MDR mouse lung carcinoma). BALB/c female mice were inoculated with 10^6^ tumor cells s.c. in the right rear footpad. Treatment given intravenously on days 7 and 14. When Dox and MLP are co-encapsulated, the doses are shown as Dox/MLP respectively. *N* mice/group = 7-9. Dose (1st → 2nd): PLD, 10 mg/kg → 8 mg/kg; PL-MLP, 35 mg/kg → 30 mg/kg; PLD+PL-MLP, 10 mg/kg + 13 mg/kg → 8 mg/kg + 10 mg/kg; PLD-MLP, 10/11 mg/kg → 8/9 mg/kg; PLAD-MLP, 10/13 mg/kg → 8/10 mg/kg. Statistical analysis (Wilcoxon matched-pairs signed rank test) of PLAD-MLP *vs*. the following groups: Untreated, *P* = 0.043; PLD-MLP, *P* = 0.004; PLD+PL-MLP, *P* = 0.005; PLD, *P* = 0.008; PL-MLP, *P* non-significant

Another tumor model tested is the M109R by subcutaneous inoculation which has a classic MDR1 phenotype^[35]^ and is much more responsive to PL-MLP than to PLD^[33]^. PLAD-MLP was again the most effective treatment [Figure 14], with greater inhibition of tumor growth than PLD, and even PLD-MLP, suggesting once again that the addition of Ald plays an important role in the therapeutic effect.

## Discussion

The experimental studies presented demonstrate the superior efficacy of a liposome co-encapsulated formulation over single drug liposomes suggesting a synergistic effect of the co-encapsulated drugs, which may have great translational value in cancer therapy. Combination therapies for the treatment of cancer are increasingly being used to address the multiple pathways of oncogenesis and cancer cell growth. Co-encapsulation in a stable nano-formulation of two or more active agents, preferably with non-overlapping toxicities and different mechanisms of action, is a unique asset of the nanomedicine platform, and a powerful tool to achieve synergistic effects and optimize drug therapy^[1]^. By spatial and time co-delivery of two drugs with otherwise different pharmacokinetic-biodistribution profiles, we can exploit combination therapy at its best and achieve optimal synergistic activity. An example is a liposome-based formulation of cytarabine and daunorubicin at an optimized 5:1 drug-to-drug ratio, known as Vyxeos®, approved for treatment of adult AML^[36]^. In this formulation the liposome carrier controls and nearly equalizes the pharmacokinetics of both drugs for the first 24 h after infusion^[37]^. There are other examples of co-encapsulated drugs in liposome and polymeric formulations demonstrating near-synchronized pharmacokinetics and positive *in vivo* results in animal models^[4,16,38]^.

Yet, the co-encapsulation approach is pharmaceutically and regulatory-wise challenging. There must be a strong rationale to justify co-encapsulation of 2 drugs in 1 nanoparticle, as opposed to co-administration of 2 nanoparticles displaying similar pharmacokinetics each with a different drug. Furthermore, combination therapy with multiple drugs delivered each by a different nanoparticle would still retain the basic pharmacologic advantage of nanoparticle-based drug delivery including controlled release rate, safe tissue distribution profile, and enhanced permeability and retention effect^[32,39]^.

In principle, one could argue that co-administration of PL-MLP and PLD could achieve the same effect that a co-encapsulated formulation does. Nevertheless, there are several advantages of the multidrug PLAD-MLP formulation:

First, it combines two well-established and valuable cancer therapy agents, MLP and Dox, with synergistic activity at well tolerated doses. The closer we get to synchronous delivery of a combination of drugs, the better the chances of an *in vivo* synergistic effect. One of the pharmacologic advantages of combining MMC with Dox is the suppression of P-glycoprotein expression that follows DNA alkylation induced by MMC. This has implication for nearly all tumor cells exposed to anthracyclines, but it is particularly important in the case of MDR+ tumor cells which can be sensitized to the cytotoxic effects of anthracyclines. Although these findings are known for more than 20 years, combination chemotherapy protocols with MMC and Dox do not have an established role in clinical practice. A major deterrent for the MMC-Dox combinations is the risk of severe toxicity^[40]^. By replacing the free drugs with a liposome formulation, we can substantially reduce toxicity, as demonstrated for PLD and PL-MLP. In studies with co-administration of PLD and PL-MLP, we found that the maximal dose of PL-MLP tolerated with a standard dose of PLD (8-10 mg/kg) is 20 mg/kg, significantly lower than the MTD of single agent PL-MLP (30-35 mg/kg). In the design of the PLAD-MLP formulation, our plan was to administer a near-full MTD of Dox (8-10 mg/kg in mice) together with a subtoxic dose of MLP (12-15 mg/kg in mice) to ensure that the treatment can be given safely and repeatedly with a similar tolerance profile to PLD. Toxicity reduction is a game changer that can open possibilities for effective and safe exploitation of the synergy between Dox and MLP, through its activation to MMC.

Furthermore, the reductive activation of the prodrug by DTT and release of MMC, as shown *in vitro*, is accompanied by release of Dox [Figure 3], suggesting that a similar process may occur *in vivo* upon contact with tissue reductive systems and help achieve simultaneous bioavailability of the main active ingredients. Based on this finding, there is a strong rationale here to expect an improved pharmacological effect when the drugs are co-encapsulated, as shown by the results of our therapeutic experiments.

Second, our pharmacokinetic studies show a unique finding with favorable implications for the multidrug formulation: the clearance of MLP from circulation is slowed down when co-encapsulated with Dox but it is not affected by concomitant administration of PL-MLP and PLD. In fact, the circulation half-life of MLP approaches that of Dox in the co-encapsulated formulation. The reason for this increased stability of MLP in PLAD-MLP is unclear. When comparing the fate of a cholesterol radiolabel and MLP, we observed that a fraction of the prodrug is cleaved from PL-MLP and cleared from circulation without evidence of liposome clearance^[19]^. Since PL-MLP and PLAD-MLP are both highly stable in plasma, we have proposed that the loss of MLP is likely the result of an interaction of liposomes with surface-bound protein disulfide isomerases in endothelial cells or platelets^[19,41]^. One characteristic of PL-MLP, which is absent in PLAD-MLP, is the presence of a significant fraction of disk-like micelles^[19]^. Since disks tend to marginate in the microcirculation flow^[42]^ and interact more readily with the endothelial blood vessel lining than standard spherical liposomes, this may, at least partly, explain the difference in MLP clearance between PL-MLP and PLAD-MLP.

An interesting and expected observation of the biodistribution studies is that once the liposome delivers its drug payload to tumor and other tissues, MLP levels go rapidly down below Dox levels. This is the result of cleavage and thiolytic activation of MLP and generation of MMC as previously demonstrated^[19]^, indicating that the liposome contents are made rapidly bioavailable in tissues.

Third, in the case of PLAD and PLAD-MLP, Ald serves as counterion to build the ammonium gradient that drives Dox inside liposomes and therefore plays a double role, as a supportive and active ingredient of the formulation. In fact, liposome encapsulation of Ald results in repurposing of Ald with evidence of immunomodulatory and antitumor activity, as reviewed recently^[6]^. Based on our results and extrapolating from our published experience with PLAD^[4]^, the use of Ald instead of sulfate as counterion for loading Dox in the PLAD-MLP formulation confers a pharmacological advantage in therapeutic effect. The rational basis for the superior therapeutic effect of PLAD-MLP over PLD-MLP, as stated previously, is the well-established immune boosting effect of aminobisphoshonates^[5,6,43]^.

As presented here, the multidrug formulation, PLAD-MLP, displayed potent *in vivo* activity in at least three mouse tumor models, outperforming the combination of PL-MLP and PLD, and using a relatively low dose of MLP equivalent to approximately 40% of the optimal dose of PL-MLP as single agent in mice (~30 mg/kg)^[19,44]^. We did not test human tumor models to ensure that the experiments are done in immunocompetent mice, since the interaction of nanomedicines with an intact immune system is very important in the therapeutic outcome particularly when an immune boosting agent such as Ald is present in the formulation^[4,45]^.

Additional advantages of PLAD-MLP for clinical translation include its ability to perform as an imaging tracer for positron emission tomography by exploiting the radiometal chelating properties of Ald and Dox^[46,47]^, and to trigger immune boosting effects mediated by Ald, particularly the potentiation of gamma-delta T cells^[48]^ and the abrogation of macrophage tumor-promoting effects^[49]^. While PLAD-MLP was not more efficacious than PLAD in some tumor models, its activity against anthracycline and multidrug resistant tumors, owing to the presence of MLP, confers a broader spectrum of activity. However, despite the attractiveness of PLAD-MLP as an all-in-one formulation, a major caveat of this approach is its complexity, and the possibility that some combinations of single drug liposomes may achieve similar therapeutic outcomes. For instance, our experiments have not covered triple single drug liposome combinations or the combination of PLAD and PL-MLP to emulate the results of PLAD-MLP which await further study.

In summary, PLAD-MLP is a unique and potent multi-drug liposome formulation for combined chemotherapy of cancer with broad spectrum antitumor activity and a safe and efficacious dose window. These results lend further support to the co-encapsulation approach for optimal pharmacological performance.

## Declarations

### Availability of data and materials

Data supporting the findings of the study can be found at the Nano-oncology Research Lab, Shaare Zedek MC, Jerusalem.

### Copyright

© The Author(s) 2022.

## References

[1] Franco MS, Oliveira MC (2019). Liposomes co- encapsulating anticancer drugs in synergistic ratios as an approach to promote increased efficacy and greater safety.. Anticancer Agents Med Chem.

[2] Tolcher AW, Mayer LD (2018). Improving combination cancer therapy: the CombiPlex(®) development platform.. Future Oncol (London, England).

[3] Lancet JE, Uy GL, Cortes JE (2018). CPX-351 (cytarabine and daunorubicin) liposome for injection versus conventional cytarabine plus daunorubicin in older patients with newly diagnosed secondary acute myeloid leukemia.. J Clin oncol.

[4] Shmeeda H, Amitay Y, Gorin J (2016). Coencapsulation of alendronate and doxorubicin in pegylated liposomes: a novel formulation for chemoimmunotherapy of cancer.. J Drug Target.

[5] Hodgins NO, Wang JT, Al-Jamal KT (2017). Nano-technology based carriers for nitrogen-containing bisphosphonates delivery as sensitisers of γδ T cells for anticancer immunotherapy.. Adv Drug Deliv Rev.

[6] La-Beck NM, Liu X, Shmeeda H, Shudde C, Gabizon AA (2019). Repurposing amino-bisphosphonates by liposome formulation for a new role in cancer treatment.. Semin Cancer Biol.

[7] Luo S, Gu Y, Zhang Y (2015). Precise ratiometric control of dual drugs through a single macromolecule for combination therapy.. Mol Pharm.

[8] Cheung RY, Rauth AM, Ronaldson PT, Bendayan R, Wu XY (2006). In vitro toxicity to breast cancer cells of microsphere-delivered mitomycin C and its combination with doxorubicin.. Eur J Pharm Biopharm.

[9] Cheung RY, Rauth AM, Yu Wu X (2005). In vivo efficacy and toxicity of intratumorally delivered mitomycin C and its combination with doxorubicin using microsphere formulations.. Anticancer Drugs.

[10] Maitra R, Halpin PA, Karlson KH (2001). Differential effects of mitomycin C and doxorubicin on P-glycoprotein expression.. Biochem J.

[11] Ihnat MA, Lariviere JP, Warren AJ (1997). Suppression of P-glycoprotein expression and multidrug resistance by DNA cross-linking agents.. Clin Cancer Res.

[12] Ihnat MA, Nervi AM, Anthony SP (1999). Effects of mitomycin C and carboplatin pretreatment on multidrug resistance-associated P-glycoprotein expression and on subsequent suppression of tumor growth by doxorubicin and paclitaxel in human metastatic breast cancer xenografted nude mice.. Oncol Res.

[13] Shuhendler AJ, O’Brien PJ, Rauth AM, Wu XY (2007). On the synergistic effect of doxorubicin and mitomycin C against breast cancer cells.. Drug Metabol Drug Interact.

[14] Shuhendler AJ, Cheung RY, Manias J, Connor A, Rauth AM, Wu XY (2010). A novel doxorubicin-mitomycin C co-encapsulated nanoparticle formulation exhibits anti-cancer synergy in multidrug resistant human breast cancer cells.. Breast Cancer Res Treat.

[15] Prasad P, Cheng J, Shuhendler A, Rauth AM, Wu XY (2012). A novel nanoparticle formulation overcomes multiple types of membrane efflux pumps in human breast cancer cells.. Drug Deliv Transl Res.

[16] Shuhendler AJ, Prasad P, Zhang RX (2014). Synergistic nanoparticulate drug combination overcomes multidrug resistance, increases efficacy, and reduces cardiotoxicity in a nonimmunocompromised breast tumor model.. Mol Pharm.

[17] Prasad P, Shuhendler A, Cai P, Rauth AM, Wu XY (2013). Doxorubicin and mitomycin C co-loaded polymer-lipid hybrid nanoparticles inhibit growth of sensitive and multidrug resistant human mammary tumor xenografts.. Cancer Lett.

[18] Gabizon A, Shmeeda H, Tahover E (2020). Development of Promitil®, a lipidic prodrug of mitomycin c in PEGylated liposomes: from bench to bedside.. Adv Drug Deliv Rev.

[19] Amitay Y, Shmeeda H, Patil Y (2016). Pharmacologic studies of a prodrug of mitomycin C in pegylated liposomes (Promitil®): high stability in plasma and rapid thiolytic prodrug activation in tissues.. Pharm Res.

[20] Golan T, Grenader T, Ohana P (2015). Pegylated liposomal mitomycin C prodrug enhances tolerance of mitomycin C: a phase 1 study in advanced solid tumor patients.. Cancer Med.

[21] Patil Y, Shmeeda H, Amitay Y, Ohana P, Kumar S, Gabizon A (2018). Targeting of folate-conjugated liposomes with co-entrapped drugs to prostate cancer cells via prostate-specific membrane antigen (PSMA).. Nanomedicine.

[22] Gabizon A, Goren D, Cohen R, Barenholz Y (1998). Development of liposomal anthracyclines: from basics to clinical applications.. J Control Release.

[23] Wei X, Patil Y, Ohana P (2017). Characterization of pegylated liposomal mitomycin c lipid-based prodrug (Promitil) by high sensitivity differential scanning calorimetry and cryogenic transmission electron microscopy.. Mol Pharm.

[24] Beheshti A, Ghaffari S, Farahani H (2019). Determination of cholesterol and its derivatives in nanoliposomes as drug delivery conveyances by HPLC-UV: a simple, accurate and cost-effective method development and validation approach.. J Chromatogr Sci.

[25] Patil Y, Amitay Y, Ohana P, Shmeeda H, Gabizon A (2016). Targeting of pegylated liposomal mitomycin-C prodrug to the folate receptor of cancer cells: Intracellular activation and enhanced cytotoxicity.. J Control Release.

[26] Gabizon A, Tzemach D, Mak L, Bronstein M, Horowitz AT (2002). Dose dependency of pharmacokinetics and therapeutic efficacy of pegylated liposomal doxorubicin (DOXIL) in murine models.. J Drug Target.

[27] Wei X, Cohen R, Barenholz Y (2016). Insights into composition/structure/function relationships of Doxil® gained from “high-sensitivity” differential scanning calorimetry.. Eur J Pharm Biopharm.

[28] Gabizon AA, Patil Y, La-Beck NM (2016). New insights and evolving role of pegylated liposomal doxorubicin in cancer therapy.. Drug Resist Updat.

[29] Peretz Damari S, Shamrakov D, Varenik M (2018). Practical aspects in size and morphology characterization of drug-loaded nano-liposomes.. Int J Pharm.

[30] Schilt Y, Berman T, Wei X, Barenholz Y, Raviv U (2016). Using solution X-ray scattering to determine the high-resolution structure and morphology of PEGylated liposomal doxorubicin nanodrugs.. Biochim Biophys Acta.

[31] Horowitz AT, Barenholz Y, Gabizon AA (1992). In vitro cytotoxicity of liposome-encapsulated doxorubicin: dependence on liposome composition and drug release.. Biochim Biophys Acta.

[32] Gabizon AA, de Rosales RTM, La-Beck NM (2020). Translational considerations in nanomedicine: the oncology perspective.. Adv Drug Deliv Rev.

[33] Gabizon AA, Tzemach D, Horowitz AT, Shmeeda H, Yeh J, Zalipsky S (2006). Reduced toxicity and superior therapeutic activity of a mitomycin C lipid-based prodrug incorporated in pegylated liposomes.. Clin Cancer Res.

[34] Gabizon A, Chemla M, Tzemach D, Horowitz AT, Goren D (1996). Liposome longevity and stability in circulation: effects on the in vivo delivery to tumors and therapeutic efficacy of encapsulated anthracyclines.. J Drug Target.

[35] Goren D, Horowitz AT, Tzemach D, Tarshish M, Zalipsky S, Gabizon A (2000). Nuclear delivery of doxorubicin via folate-targeted liposomes with bypass of multidrug-resistance efflux pump.. Clin Cancer Res.

[36] Alfayez M, Kantarjian H, Kadia T, Ravandi-Kashani F, Daver N (2020). CPX-351 (vyxeos) in AML.. Leuk Lymphoma.

[37] Feldman EJ, Lancet JE, Kolitz JE (2011). First-in-man study of CPX-351: a liposomal carrier containing cytarabine and daunorubicin in a fixed 5:1 molar ratio for the treatment of relapsed and refractory acute myeloid leukemia.. J Clin Oncol.

[38] Zhang RX, Cai P, Zhang T (2016). Polymer-lipid hybrid nanoparticles synchronize pharmacokinetics of co-encapsulated doxorubicin-mitomycin C and enable their spatiotemporal co-delivery and local bioavailability in breast tumor.. Nanomedicine.

[39] Golombek SK, May JN, Theek B (2018). Tumor targeting via EPR: strategies to enhance patient responses.. Adv Drug Deliv Rev.

[40] Doyle LA, Ihde DC, Carney DN (1984). Combination chemotherapy with doxorubicin and mitomycin C in non small cell bronchogenic carcinoma: Severe pulmonary toxicity from q 3 weekly mitomycin C.. Am J Clin Oncol.

[41] Flaumenhaft R, Furie B (2016). Vascular thiol isomerases.. Blood.

[42] Toy R, Hayden E, Shoup C, Baskaran H, Karathanasis E (2011). The effects of particle size, density and shape on margination of nanoparticles in microcirculation.. Nanotechnology.

[43] Zocchi MR, Tosetti F, Benelli R, Poggi A (2020). Cancer nanomedicine special issue review anticancer drug delivery with nanoparticles: extracellular vesicles or synthetic nanobeads as therapeutic tools for conventional treatment or immunotherapy.. Cancers (Basel).

[44] Gabizon A, Amitay Y, Tzemach D, Gorin J, Shmeeda H, Zalipsky S (2012). Therapeutic efficacy of a lipid-based prodrug of mitomycin C in pegylated liposomes: studies with human gastro-entero-pancreatic ectopic tumor models.. J Control Release.

[45] Rios-Doria J, Durham N, Wetzel L (2015). Doxil synergizes with cancer immunotherapies to enhance antitumor responses in syngeneic mouse models.. Neoplasia (New York, NY).

[46] Edmonds S, Volpe A, Shmeeda H (2016). Exploiting the metal-chelating properties of the drug cargo for in vivo positron emission tomography imaging of liposomal nanomedicines.. ACS Nano.

[47] Gawne P, Man F, Fonslet J (2018). Manganese-52: applications in cell radiolabelling and liposomal nanomedicine PET imaging using oxine (8-hydroxyquinoline) as an ionophore.. Dalton Trans.

[48] Man F, Lim L, Volpe A (2019). In Vivo PET tracking of (89)Zr-Labeled Vgamma9Vdelta2 T cells to mouse xenograft breast tumors activated with liposomal alendronate.. Mol Ther.

[49] Rajan R, Sabnani MK, Mavinkurve V (2018). Liposome-induced immunosuppression and tumor growth is mediated by macrophages and mitigated by liposome-encapsulated alendronate.. J Control Release.

