# Supplementary material for: Liposome co-encapsulation of anti-cancer agents for pharmacological optimization of nanomedicine-based combination chemotherapy
Source: Cancer Drug Resist. 2021 Jun 19;4(2):463–84. doi: 10.20517/cdr.2020.87 (PMC9019273; doi:10.20517/cdr.2020.87)

Supplementary Data: Gabizon et al.

HPLC protocol for analysis of liposome co-encapsulated Dox and MLP:

Column: Phenomenex Hyperclone 5μ BDS C18 130 A 150 x 4.60 mm 5 micron

(+ guard)

Cat # 00F-4420-E0

Security guard Cartridges C18 4 x 3.0 mm Cat # XAJO-4287

Security guard holder kit XKJO-4282

Mobile phase: gradient

Time (min)	A (%)	B (%)
0	100	0
12	100	0
12.1	0	100
25.0	0	100
25.1	100	0
30.0	100	0

A = MeOH/ NaH_2PO_4 10 mM pH=6 70/30

B = MeOH/IPA 70/30

Flow: 1 ml/min

Wavelength: 1-10 min 480 nm

10-30 min 360 nm

Sample volume = 20 ul

Run time: 30 min

Retention Time = DOXORUBICIN 3.7 min; MLP 20.8 min

Preparation of a standard curve for DOXORUBICIN

Prepare stock solution of Doxorubicin 1 mg/ml in distillated water with 5% Dextrose.

Dilute the stock solution in SDS10% to obtain concentrations of 500, 40 and 20μg/ml.

Dilute the solution of 500 μg/ml in SDS10% to 5 and 10 μg/ml.

Heat the solutions at 60 °C for 5 min. Filter them in 0.2 Nylon spin-X centrifuge tube for 3 min at 10000 rpm. Run in the HPLC and do a standard curve from 5-40 μg/ml.

Preparation of a standard curve for MLP

Prepare stock solution of MLP 2 mg/ml in IPA.

Dilute the stock solution in SDS10% to obtain concentrations of 10, 20, 40 and 80μg/ml.

Vortex and heat the solutions at 60 °C for 5 min. Filter them in 0.2 Nylon spin-X centrifuge tube for 3 min at 10000 rpm. Run in the HPLC and do a standard curve from 10-80 μg/ml.

Preparation of PLAD-MLP for HPLC injection

Dilute the liposomes 1/50 in SDS 10%, vortex and heat at 60 °C for 5 min. Filter them in 0.2 Nylon spin-X centrifuge tube for 3 min at 10000 rpm and run in the HPLC in duplicates under the conditions described above.

Supplementary Data: Table S1: CYTOTOXICITY SUMMARY of IC50's

Cell Line	iGrov-1		N-87		T-24		M109R		NCI/ADR	
**IC50 (μM)**	**DOX**	**MMC /MLP**	**DOX**	**MMC /MLP**	**DOX**	**MMC /MLP**	**DOX**	**MMC /MLP**	**DOX**	**MMC /MLP**
Free MMC		0.7		0.18		0.05		0.3		0.4
Free Dox	1.1		0.19		0.1		8.7		2.5	
PLD	16.4		2.7		2.95		>25		>25	
PL-MLP		18.3		16.3		17.4		>25		>25
PL-MLP +PLD	12.1	10	4.3	3.6			>25	>25	>25	>25
**PLAD-MLP**	**3.4**	**2.8**	**0.5**	**0.4**	**0.6**	**0.5**	**8.22**	**19.5**	**7.8**	**6.6**

Supplementary data, Figure S1: : *In vitro* comparative cytotoxicity in T24 cells: PLD-MLP and PLAD-MLP are almost equally cytotoxic and significantly more cytotoxic than PL-MLP and PLA-MLP.

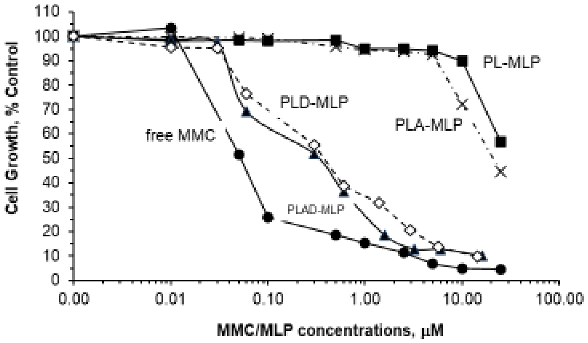

<u>**Supplementary data Fig S2**</u>:
Toxicity study: Balb/C Female mice injected i.v. on days 1, 7 and 21 with PLAD-MLP (n=4), or with PLD-MLP (n=4) at a dose of 10 mg/kg (n=4) based on Dox concentration.

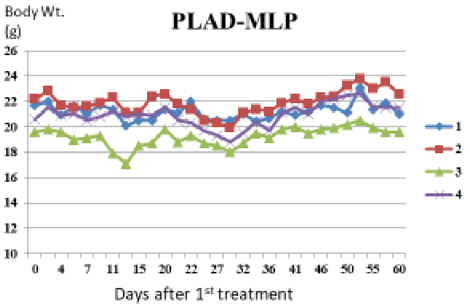

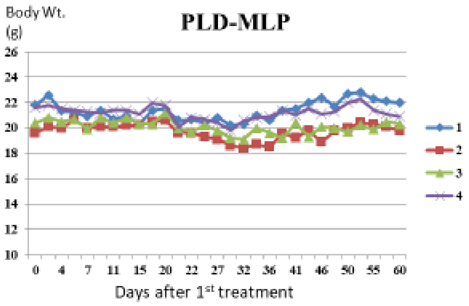

Supplementary data Fig. S3:
PLAD is as effective as PLAD-MLP in the 4T1 tumor model. Other technical details as described on legend to Fig. 11.

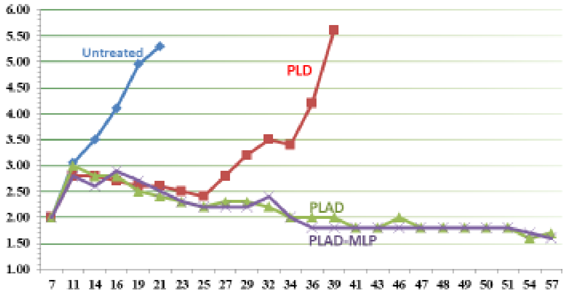

Supplement: Supplementary file 1 [file cdr-4-463-SupplementaryMaterials.pdf]
